# A Year at the Forefront of Streptophyte Algal Evolution

**DOI:** 10.1242/bio.061673

**Published:** 2024-09-19

**Authors:** Alexander M. C. Bowles

**Affiliations:** Department of Biology, University of Oxford, Oxford OX1 3SZ, UK

**Keywords:** Streptophytes, Algal evolution, Plant terrestrialisation

## Abstract

Land plants originated from an algal ancestor ∼500 million years ago in one of the most important evolutionary events for life on Earth. Extant streptophyte algae, their closest living relatives, have subsequently received much attention to better understand this major evolutionary transition. Streptophyte algae occupy many different environments, have diverse genomes and display contrasting morphologies (e.g. unicellular, filamentous, three-dimensional). This has historically made inferring these evolutionary events challenging. This A Year at the Forefront Review focusses on research published between July 2023 and June 2024 and intends to provide a short overview of recent discoveries, innovations, resources, and hypotheses regarding streptophyte algal evolution. This work has provided mechanistic insights into ancient evolutionary events that prefigured the origin of land plants and raises new questions for future research into streptophyte algae.

## Introduction

**Fig. 1.**
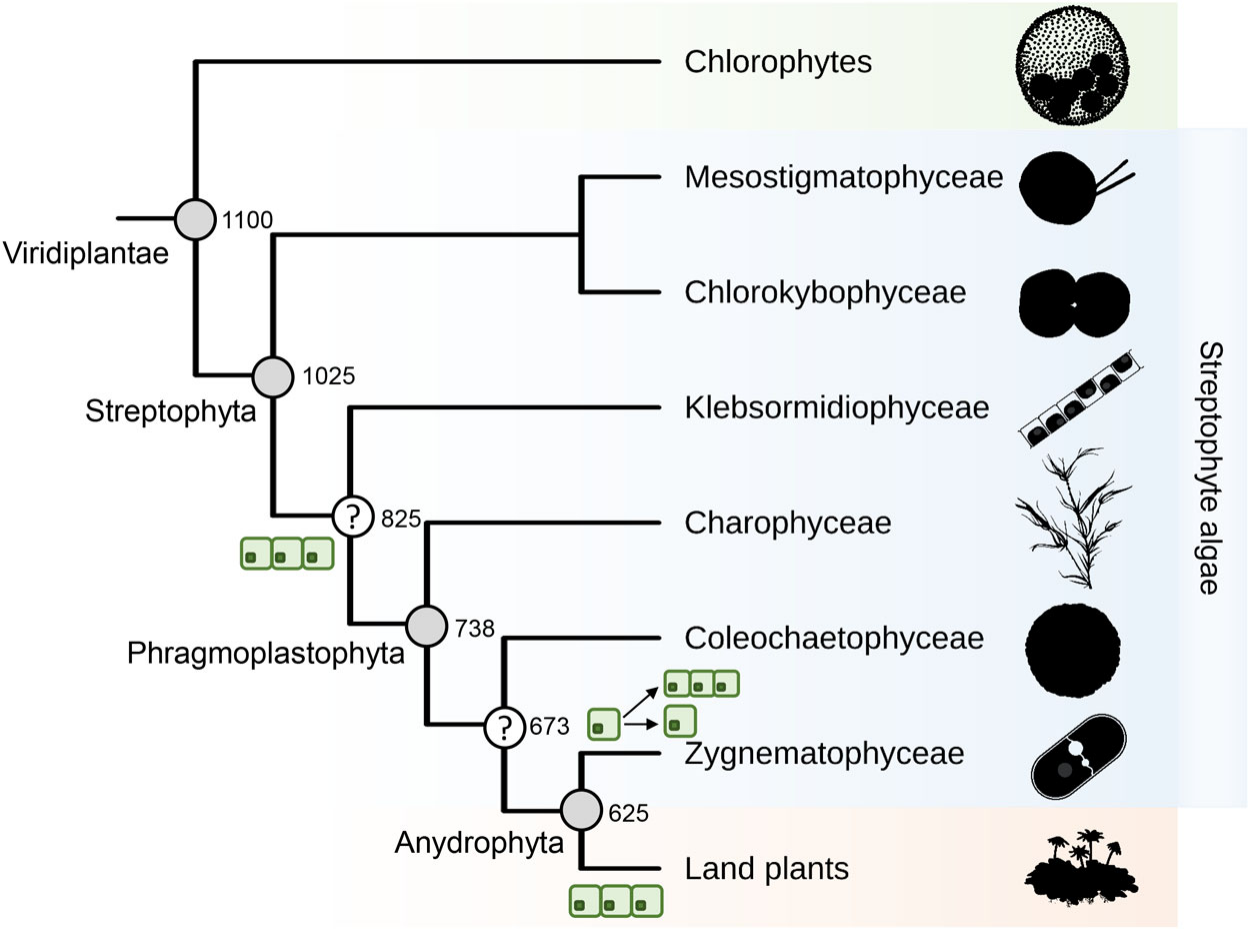
**The evolution of streptophytes.** A cladogram of the current understanding of streptophyte relationships. Taxa are highlighted in orange (land plants), blue (streptophyte algae) and green (chlorophyte algae). The origin of multicellularity as well as its loss and multiple independent regains in Zygnematophyceae are highlighted. Nodes with no commonly used nomenclature are highlighted with a question mark. Numbers at each internal node highlight divergence time in millions of years, with estimates derived from Harris et al. (2022). Silhouettes are sourced from Phylopic, with credit to Matt Crook (https://creativecommons.org/licenses/by-sa/3.0/).

The origin of land plants from a streptophyte algal ancestor is considered to be one of the major transitions in evolution (Bowles et al., 2023; de Vries and Archibald, 2018; Donoghue et al., 2021). Extant streptophyte algae form six distinct groups, representing over 5000 species, with those in the Charophyceae the most morphologically complex (Bierenbroodspot et al., 2024a). Comparisons of streptophyte morphology and early phylogenetic studies predicted Charophyceae and Coleochaetophyceae as the most likely sister group to land plants (Graham et al., 2000; Karol et al., 2001). Plant fossils, acting as evolutionary intermediates, can add weight to inferences made from living plants. However, the streptophyte fossil record is sparse, with only a small number of fossil spores and algae (e.g. *Paleonitella*, *Rhyniotaenium*) previously identified (Edwards and Lyon, 1982; Krings, 2022; Strother and Foster, 2021). Leveraging the One Thousand Plant Transcriptome project, large-scale phylogenetic analysis identified the unicellular Zygnematophyceae, rather than the morphologically complex Charophyceae, as the sister group to land plants (Puttick et al., 2018; Wickett et al., 2014). With this robust phylogeny (Fig. 1), researchers have begun to piece together the myriad of clues left behind to understand the molecular and morphological innovations of ancient streptophytes.

Comparative genomics and transcriptomics have started to reveal the extent to which successive streptophyte ancestors were pre-adapted for life on land, including adaptations to UV light stress, drought and gravity (Cheng et al., 2019; de Vries et al., 2018; Hori et al., 2014; Jiao et al., 2020; Liang et al., 2019; Wang et al., 2019). Evolutionary developmental studies have also identified conserved and novel gene functions in land plants and their close algal relatives, involved in three-dimensional (3D) growth (Moody et al., 2018, 2021), phytohormone signalling (Ju et al., 2015) and multicellular development (Mulvey and Dolan, 2023). Analysis of taxa across the tree of life has illuminated the role of horizontal gene transfer from fungi and bacteria in aiding this major transition (Cheng et al., 2019; Delaux et al., 2015; Ma et al., 2022; Shinohara and Nishitani, 2021). Temporal analyses have demonstrated the timescale of streptophyte evolution spanning the Meso-Neoproterozoic eras to the present day (del Cortona et al., 2020; Harris et al., 2022; Morris et al., 2018; Strassert et al., 2021). Combining this information with analysis of environmental variables has built a picture of the temperature (Hearing et al., 2018), O_2_ and CO_2_ levels (Brocks et al., 2017) present during streptophyte evolution, as well as the interaction between algal ancestors and their environment.

These different strands of evidence have helped us understand how the first land plants evolved. Here, I review several important advances over the last twelve months in the field of streptophyte algal evolution. With the brief nature of this piece, I have selected a small collection of papers to highlight. However, absence of mention does not suggest lack of importance or significance.

## Discoveries

Like many fields, omics data have driven new insights into streptophyte algae, particularly the evolution of stress tolerance (e.g. Cheng et al., 2019; Jiao et al., 2020; Wang et al., 2019). Recently, genome and transcriptome sequencing of Zygnematophyceae species has revealed an expanded gene repertoire for stress tolerance that predates plant terrestrialisation (Feng et al., 2024; Rieseberg et al., 2024 preprint). For example, gene novelties in the common ancestor of land plants and Zygnematophyceae led to the origin of major enzymes involved in cell wall biosynthesis and re-modification, while duplicated genes were associated with signalling cascades and environmental responses (Feng et al., 2024). Furthermore, transcriptomics of two Zygnematophyceae species and the moss *Physcomitrium patens* revealed shared genetic hubs for the synthesis of apocarotenoids, which are involved in physiological responses to osmotic stress (Rieseberg et al., 2024 preprint). Coupling experimental desiccation with transcriptomics also revealed conserved stress responses likely common to the ancestor of Zygnematophyceae, linked to plastid biology, amino acid pathways and metabolism (Rieseberg et al., 2023a). Lineage-specific responses, and therefore likely not found in the ancestor of Zygnematophyceae, were seen in photobiology and the length of desiccation tolerance. Responses to UV radiation in a zygnematophyte (*Serritaenia testaceovaginata*) were observed with experimental transcriptomics suggesting the conserved synthesis of a polyphenolic sunscreen pigment, resembling plant lignin (Busch et al., 2024). Furthermore, the discovery of bona fide phenylalanine ammonia lyase in streptophytes (Schwarze and Petersen, 2024) highlights the deep evolutionary origins of the phenylpropanoid pathway (de Vries et al., 2021). Together, these new works highlight the selective pressures of abiotic stresses associated with terrestrial environmental conditions.

Phytohormones are linked to environmental stress responses (e.g. abscisic, salicylic and jasmonic acid) (Jia et al., 2023; Rieseberg et al., 2023b), as well as many aspects of developmental regulation (Hernández-García et al., 2021; Mutte et al., 2018; Powell and Heyl, 2023). Previously, the origin of phytohormone biosynthesis and signalling pathways have been shown to either predate or accompany the land plant transition (Bowles et al., 2020). However, the extent to which these compounds are produced in streptophyte algae remained uncertain. Experimental analysis of green plants, including all lineages of streptophyte algae, demonstrated that auxin, salicylic acid, ethylene and some forms of cytokinin are common to Viridiplantae (Schmidt et al., 2024). Some streptophyte algae produce jasmonates and abscisic acid whereas these phytohormones are found in all land plants. Conversely, some forms of cytokinin and auxin were specific to land plants. This analysis depicts a stepwise evolution of developmental and environmental regulation via phytohormone signalling across streptophytes.

Accompanying these analyses into stress tolerance are insights into streptophyte trait evolution. For example, the last common ancestor of Phragmoplastophyta and Klebsormidiophyceae has been identified as likely multicellular (Bierenbroodspot et al., 2024b; Bowles et al., 2024a). Further transcriptomic analysis has identified complex genome evolution linked with the origin of this first multicellular streptophyte (Bierenbroodspot et al., 2024b). Ancient environmental pressures of the Cryogenian (e.g. extreme cold) have been proposed as drivers of multicellularity in streptophytes (as well as in other archaeplastid algae, animals and fungi; Simpson, 2021). The plausibility of this hypothesis for the first multicellular streptophyte was recently confirmed, using ancestral state reconstruction on a time-calibrated phylogeny (Bowles et al., 2024a). Another study, using the model green alga *Chlamydomonas reinhardtii*, further demonstrated that experimental simulation of Cryogenian snowball Earth environments led to the development of multicellular algae (Halling et al., 2024 preprint). Though multicellularity evolved early during streptophyte evolution, extant algae have diverse morphological forms (Domozych et al., 2016). For example, the ancestor of Zygnematophyceae underwent reductive morphological evolution, converting from multicellular to unicellular (Bowles et al., 2024a; Hess et al., 2022). Comparative genomics revealed that this transition was accompanied by reductive genome evolution (Bowles et al., 2024b). In addition, land plants, like other complex multicellular groups (e.g. animals, fungi), exhibit an evolutionary burst of phenotypic disparity (Clark et al., 2023).

## Technological innovations

Though comparative genomics and phylogenetics can infer much about the origin of genes, genetic manipulation is needed to fully unravel functions conserved across land plants and their algal relatives. A new electroporation-based transformation protocol for the model Zygnematophyceae species, *Penium margaritaceum*, has been developed that will enable a better understanding of gene function, particularly for those important in stress tolerance (Carrillo-Carrasco et al., 2023). This approach has also been shown to successfully deliver proteins in other zygnematophycean species (e.g. *Closterium sp.*, *Mesotaenium endlicherianum*), allowing for comparisons between the two major groups of Zygnematophyceae, the Desmidiales and Zygnematales.

Innovative electron microscopy imaging and 3D-reconstruction have advanced our understanding of algal conjugation, zygospores and cell wall structures, adaptations that are vital for terrestrial habitats (Permann et al., 2023). Focused ion beam scanning electron microscopy (FIB-SEM) provided insights into the cytological organisation of *Zygnema vaginatum* whilst transmission electron microscopy (TEM) identified thin cell walls in younger zygospores and tripartite walls in mature algal cells (Permann et al., 2023). These techniques offer great potential for identifying commonalities of cell wall structures shared between streptophyte algae and land plants, thereby aiding our understanding of the mechanisms that facilitated the evolutionary transition from water to land.

## New resources

The four *Zygnema* genomes, discussed above, are a key resource for comparative genomics (Feng et al., 2024) and represent the first chromosome-scale genome assemblies for any streptophyte algae. Furthermore, additional transcriptome data for Zygnematophyceae, coupled with time series experimental profiling, offers unparalleled insight into stress tolerance capabilities that predate the origin of land plants (Rieseberg et al., 2024 preprint). Within the Zygnematophyceae, a metagenome-assembled genome of the cold tolerant glacier alga *Ancylonema nordenskioldii* has also provided insight into streptophyte evolution in extreme environments (Bowles et al., 2024b). These uniquely adapted algae have previously only been sequenced using marker-based analysis (Procházková et al., 2021). Improved gene annotation for *Mesotaenium endlicherianum* (Dadras et al., 2023) and genome sequencing of *Closterium sp.* (Sekimoto et al., 2023) provides further insight into zygnematophyte evolution.

To understand the evolution of Klebsormidiophyceae, 24 new transcriptomes were sequenced (Bierenbroodspot et al., 2024b). These data and the new phylogenetic framework are an important resource to understand the evolution of multicellularity in streptophytes.

## New hypotheses

The work above has highlighted several interesting areas for future research, concerning terrestrial habitation, multicellularity and omics data. The first concerns the environmental conditions of streptophyte algal evolution, spanning the late Mesoproterozoic–Neoproterozoic (1200-540 million years ago). Recent work investigated hypotheses suggesting that adaptations of modern-day streptophyte glacier algae represent a remnant of an ancient Cryogenian adaptation (Williamson et al., 2019; Žárský et al., 2022). The adaptations of glacier algae to surface ice environments (Millar et al., 2024) appear to have evolved more recently, but the physical environment was clearly a significant driver of streptophyte evolution (Bowles et al., 2024b). The exact nature of this is yet to be determined.

Multicellularity appears to be labile across early diverging streptophytes (Bierenbroodspot et al., 2024b; Bowles et al., 2024b). Interestingly, multicellular development genes are found in single-celled algae, enabling both forward, and reverse, evolutionary trajectories. This is one of a handful of examples in the eukaryotic tree of life of the reductive evolution of multicellular to unicellular organisms. Rather than being a novelty of land plants, this suggests multicellularity in streptophytes is much older (Paps et al., 2023). The molecular and regulatory underpinning of this major transition is still unclear.

Coleochaetophyceae is the sister lineage to Anydrophyta, the group containing Zygnematophyceae and land plants (Fig. 1; Wickett et al., 2014). It is multicellular, sitting morphologically between the complex three-dimensionality of Charophyceae and filamentous nature of Zygnematophyceae. Though transcriptomic data has been produced (Leebens-Mack et al., 2019), no genome has yet been published for Coleochaetophyceae. This is a key resource to further understand streptophyte algal evolution.

## Future prospects

With a well-resolved backbone phylogeny, a key future objective is identifying the synapomorphies of streptophyte algal groups and using these to assign informative names. Currently, the major groupings are Streptophyta, incorporating all streptophyte algae and land plants, Phragmoplastophyta, which includes all streptophytes that complete mitosis via a phragmoplast (Charophyceae, Coleochaetophyceae, Zygnematophyceae and land plants) and Embryophyta, commonly known as land plants. Distinguishing the key features of other streptophyte groups is needed to understand this major evolutionary transition and derive useful names for algal ancestors. For example, collectively Embryophyta and Zygnematophyceae have recently been coined Anydrophyta, due to the evolution of molecular mechanisms to cope with drought (Rensing, 2020). There are several other groups of streptophyte algae without names (Anydrophyta and Coleochaetophyceae as well as Phragmoplastophyta and Klebsormidiophyceae), which acts as a barrier when discussing novel evolutionary innovations.

New technologies and taxonomically important omics resources will offer important opportunities for future research into streptophyte algal evolution. In particular, this will reveal the extent to which land plants evolved through stepwise evolution (rather than emerging onto land in a single evolutionary step), gaining (pre)adaptations along the backbone of the streptophyte phylogeny. Another technology, not yet discussed, is the advent of single cell sequencing. For land plants, this has provided important insights into root and phloem development in detailed resolution (Dorrity et al., 2021; Kim et al., 2021; Otero et al., 2022; Ryu et al., 2019). This technology could aid our understanding of the evolution of multicellular development in streptophyte algae and for sequencing unculturable taxa.

Over the last 12 months, research has answered fundamental questions about the evolutionary adaptations shared between land plants and their closest relatives as well as characteristics that are unique to streptophyte algae. New resources, in the form of genomic, transcriptomic and morphological datasets, have improved the taxonomic resolution of sparsely sampled algae. Technical innovations, in imaging and transformation protocols, have paved the way for new forms of analysis into cell wall structure and gene function, respectively. Key discoveries regarding environmental distribution, stress tolerance and morphological diversity have aided our understanding of streptophyte algal evolution. This last year's research has laid important groundwork and raised exciting questions for the future.
